# A Pilot Study of the Effects of Individualized Home Dual Task Training by Mobile Health Technology in People with Dementia

**DOI:** 10.3390/ijerph20085464

**Published:** 2023-04-11

**Authors:** Eduardo Villamil-Cabello, Mercedes Meneses-Domínguez, Ángela Fernández-Rodríguez, Patricia Ontoria-Álvarez, Alfonso Jiménez-Gutiérrez, Miguel Fernández-del-Olmo

**Affiliations:** 1 Centre for Sport Studies, Rey Juan Carlos University, 28943 Madrid, Spain; e.villamil.2021@alumnos.urjc.es (E.V.-C.); alfonso.jimenez@urjc.es (A.J.-G.); 2 GO fitLAB, Ingesport, 28003 Madrid, Spain; 3 AFA Coslada, 28822 Madrid, Spain; afacorredordelhenares@gmail.com; 4 Instituto Cántabro de Servicios Sociales, 39006 Cantabria, Spain; 5 Servicio Cántabro de Salud, 39011 Cantabria, Spain; patriciacristina.ontoria@scsalud.es

**Keywords:** mobile health technology, dual-task, homebase training

## Abstract

The objective of this pilot study was to evaluate the effects of dual-task training implemented by mobile health technology on performance on motor and dual-task tests in subjects with dementia. Nineteen subjects with a medical diagnosis of dementia were assigned to an experimental group (EG, *n* = 12) or control group (CG, *n* = 7). The EG participated in 24 sessions (3/week) of a homebase dual-task exercises program, in addition to their ongoing cognitive and physiotherapy treatment. The training program was implemented individually in the patient’s home by caregivers or relatives through electronic devices controlled by a mobile application. Before (Pre) and after (Post) the program, performance on motor and motor/cognitive (dual-task) tests were evaluated. Motor evaluation included gait at preferred and maximal speed, the Up and Go, and the Handgrip Strength test. Dual-task tests included gait with subtraction 3 s from 100 and naming animals (verbal fluency). The CG only performed the evaluations in addition to their cognitive and physiotherapy treatment. The statistical analysis (ANOVA Group*Test) showed a statically significant improvement for both dual-task tests in the EG after the training program, while the CG showed an impairment in the verbal fluency test. Conclusion: the implementation of a home exercise program carried out with mobile technology in people with dementia is feasible and positively affects their performance on dual tasks.

## 1. Introduction

According to the World Health Organization, dementia is a syndrome—usually of a chronic or progressive nature—that leads to deterioration in cognitive function beyond what might be expected from the usual consequences of biological ageing. It affects memory, thinking, orientation, comprehension, calculation, learning capacity, language, judgement, and has a severe impact on activities of daily living (ADL). Currently more than 55 million people live with dementia worldwide. In the future, with increasing life expectancy, the number of people experiencing dementia will increase dramatically, with an estimated nearly 10 million new cases every year. This will not only have an impact on the quality of life of patients, but will increase the burden on family caregivers [1], community care and health services [2].

Exercise has been suggested as a potential lifestyle factor in reducing or delaying the progression of the symptoms of dementia [2]. However, establishing the most recommended type of exercise for people with dementia is a great challenge given the wide heterogeneity of the motor and cognitive symptoms associated with dementia. Nevertheless, several recent meta-analyses show that physical activity and exercise can improve cognition in elderly with Alzheimer disease (AD) [3,4]. Furthermore, cognitive intervention and physical exercise combined may produce additive and synergistic effects in older adults with dementia, resulting in greater cognitive benefits than cognitive training or physical exercise alone [5]. In addition to exercise and cognitive training, sensory and multisensory stimulations can effectively ameliorate the pathology of AD, arouse memory, and improve cognition and behaviors [6]. There is evidence that exercise increases the volume of the hippocampus [7] and prefrontal cortex [8], and may potentiate neurogenesis [9], while sensory stimulation could modulate neural oscillations [10] and enhance brain plasticity [11].

In addition to the type of exercise, there are also two highly relevant issues when we want to implement, in a real context, an exercise program in subjects with dementia: (i) to what degree the cognitive or even motor improvements reported in numerous studies translate into an improvement in the activities of daily life; and (ii) how does socioeconomic status condition the patient’s access to the exercise program?

Regarding the first point, it is well known that on many occasions the improvements obtained by an intervention are limited to the condition of the laboratory [12]. Therefore, numerous studies have explored the effect of exercise and cognitive training using the dual task-walking paradigm. In fact, dual task walking performance is one of the main goals and outcomes of rehabilitation for individuals with neurological disorders [13]. This is not surprising due to the relevance of the double-task walk for the individual’s daily ambulation and, therefore, their motor autonomy and integration in society [14]. In addition, divided attention—the ability to respond to multiple stimuli simultaneously—is frequently affected more than other domains, such as sustained attention [15]. Therefore, dual-task ability is an example of cognitive control where the prefrontal cortex plays the main role [16].

In relation to socioeconomic status, health systems of many countries do not cover the expenses associated with the interdisciplinary treatment that dementia requires, making the economic status of the patient or his family a differential factor in the treatment of this and other conditions [17]. This is even more relevant considering the higher prevalence of dementia in people with low socioeconomic status [18,19]. For example, in Spain, a large part of the care for dependent people is provided by people in the family environment who are not linked to any professional care service [20]. Due to this reason, it is common for relatives of people with dementia to create local associations in which to implement, through self-financing, different therapeutic interventions as an alternative to the generally saturated health systems [1,21]. These associations provide therapeutic treatments to patients with a wide spectrum of involvement, and even with different diagnoses of dementia. This is a challenge from the therapeutic point of view since often the physiotherapy treatment is group-based, making it difficult to individualize the exercises according to the features of each patient [22]. In addition, this associative movement cannot meet the needs of patients who live in highly depopulated rural areas or who simply cannot travel to the association to participate in the exercise program [23,24]. The development of telemedicine, electronic devices and mobile health applications could be an interesting option for those patients who find themselves in the situations mentioned above. However, there are several limitations regarding the use of mobile health apps, such as that less than 1% of them are grounded in research evidence [25].

The objective of this pilot study is to evaluate the effects of dual-task training, characterized by a combination of physical, cognitive and sensory exercises performed simultaneously, on performance on dual gait tasks in subjects with dementia. The training program will be implemented individually in the patient’s home by caregivers or relatives through electronic devices controlled by a mobile application. Therefore, a second objective of the study is to explore the feasibility and effectiveness of this type of technology in the treatment of people with dementia.

## 2. Materials and Methods

This is a pilot study from a registered clinical trial (ClinicalTrials.gov Identifier: NCT05295966). This study was conducted in full compliance with the Declaration of Helsinki 1964 (updated in Fortaleza, 2013) and approved by the Local Ethics Committee.

### 2.1. Study Participants

**Figure 1:**
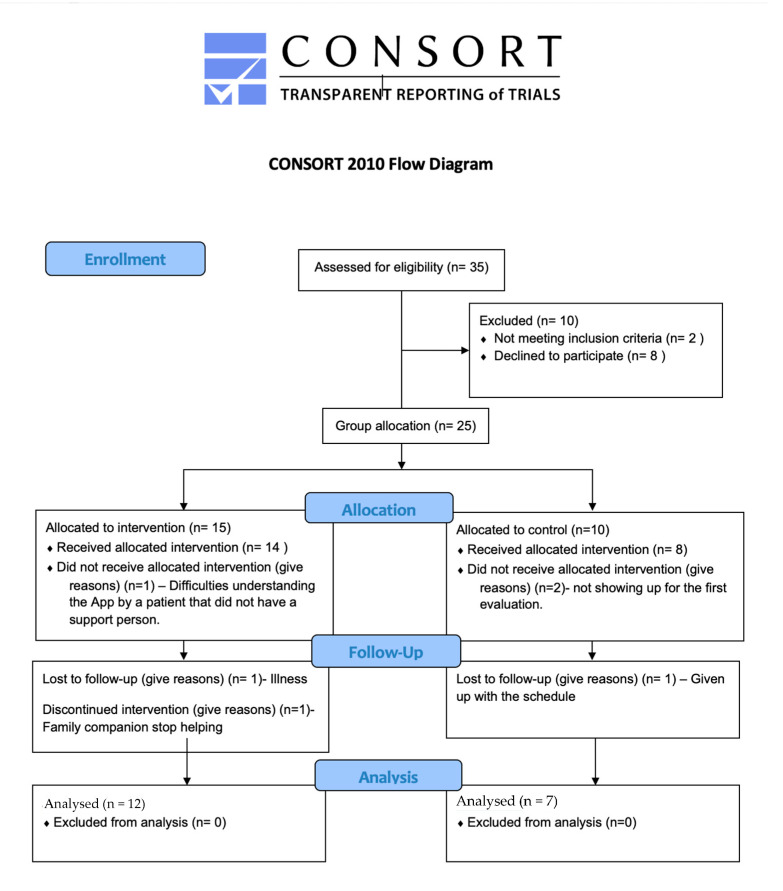
Flow diagram of the subject’s recruitment and participation. Inclusion requirements: (i) have a support caregiver willing to accompany participants to assessment visits as well as during training sessions; (ii) adequate visual and auditory ability to interact with the electronic training devices; and (iii) ability to participate in all scheduled assessments and exercise programs. Exclusion criteria included: (i) clinically evident stroke with severe motor impairment; (ii) myocardial infarction or coronary artery disease; (iii) uncontrolled hypertension; and (iv) musculoskeletal symptoms that would make exercise difficult.

Thirty five subjects with a medical diagnosis of cognitive impairment were recruited in 2022 from several local Alzheimer associations in Madrid’s community, Spain, and they were assessed for eligibility (Figure 1).

Participants were assigned to an experimental group (EG) or control group (CG) according to the willingness of the participant’s relatives or caregivers to implement the intervention program at home. All the participants of the experimental group and their caregivers attended a face-to-face meeting where the operation of the application and the electronic devices were explained to them. This session was also used to check that all patients had sufficient perceptual skills (visual, auditory, and proprioceptive) to interact correctly with the devices and that they understood the instructions of some of the exercises designed. Otherwise, they would be suggested to join the control group. For example, patients had to touch the devices based on the color of the light and/or sound, and also passively detect the vibration of the device. One patient was excluded from the study due to difficulties in using the mobile application. In this case, the patient did not have a support person (family member or caregiver) to carry out the intervention. The other patients were able to interact satisfactorily with the electrical devices.

A total of 19 subjects, 12 in the EG and 7 in the CG, completed the evaluations and training sessions, and were included in the statistical analysis. Table 1 shows the scores of each participant in the Mini-Mental State Examination (MMSE), the Lawton Instrumental Activities of Daily Living Scale (Lawton IADL) and the Barthel Scale/Index (BI). The MMSE is a 30-point questionnaire that is used extensively in clinical and research settings to measure cognitive impairment [26,27]. Any score of 24 or more (out of 30) indicates a normal cognition. The raw score may also need to be corrected for educational attainment and age [28]. Even a maximum score of 30 points can never rule out dementia, and there is no strong evidence to support this examination as a stand-alone one-time test for identifying high risk individuals who are likely to develop Alzheimer’s. [26] The Lawton IADL is a self-report scale that assesses eight tasks providing information about functional skills necessary to live independently in the community with a summary score from 0 (low function) to 8 (high function) [29]. The BI is an ordinal scale used to measure performance on activities of daily living. The Barthel Index measures the degree of assistance required by an individual on 10 items of mobility and self-care ADL [30]. A higher number is associated with a greater likelihood of being able to live at home with a degree of independence following discharge from hospital.

### 2.2. Procedure

The EG participated in 24 sessions (3/week) of a home-based cognitive-multisensory-physical exercise program. Before (Pre) and after (Post) the program, performance on motor and motor/cognitive (dual-task) tasks were evaluated. The CG only performed the evaluations. All the participants maintained their ongoing cognitive and physical rehabilitation programs at their local associative center.

### 2.3. Motor and Dual-Task Assessments

The patients performed the tests in the following order: (i) Gait at preferred speed; (ii) Gait dual-task tests; (iii) Gait at maximal speed; (iv) Timed Up and Go test (TUG); (v) Handgrip Strength.

#### 2.3.1. Gait at Preferred Speed

The patients walked the length of a room at their preferred speed, covering a distance of 8 m with round-trip trajectories until completing a total time of one minute. The test began with the indication “whenever you want you can start walking”. The temporal gait parameters were recorded using an optical detection system (Optogait, Microgait, Mahopac, NY, USA). This optical and modular system included transmitting and receiving bars of infrared LEDs displayed in parallel along 5 m of a 9 m walkway. Each bar contained 100 LEDs. When subjects entered the area limited by the bars, their feet blocked the transmission and reception, and the spatiotemporal gait data was transferred to a personal computer at a sample rate of 1000 HZ. The OptoGait System has strong concurrent validity along with relative and absolute test-retest reliabilities [31]. The main outcome measure was the speed (*m*/*s*), calculated as the average speed across the one-minute walk.

#### 2.3.2. Gait Dual Task

We conduct two dual-task conditions, subtraction and a verbal fluency task. For the dual-task trials, participants walked for 1 min while subtracting serial 3 s from 100 aloud, and naming animals aloud. We chose those dual-task conditions based on previous research which demonstrated that naming animals depends more on verbal fluency and semantic memory, while subtraction relies on working memory and attention [32,33]. The instructions for the dual-task test did not prioritize either the gait or cognitive task, in order to better simulate a daily living activity. Dual-task interference was calculated as speed reduction during the dual task in comparison with the gait at preferred speed as follows [34]:

$$\mathrm{Dual}-\mathrm{task}\;\mathrm{interference}\;=\frac{-\left(\mathrm{dual}\;\mathrm{task}\;\mathrm{gait}\;\mathrm{speed}\;-\;\sin\mathrm{gle}\;\mathrm{task}\;\mathrm{gait}\;\mathrm{speed}\right)}{\sin\mathrm{gle}\;\mathrm{task}\;\mathrm{gait}\;\mathrm{speed}}\times 100\%$$

Therefore, higher scores of dual-task interference values indicate a bigger impact of the cognitive task over the gait performance. 

#### 2.3.3. Gait at Maximal Speed

The same protocol was carried out as in the “Walk at preferred speed” test, but in this case the instruction was to walk at the maximum possible speed.

#### 2.3.4. Timed Up and Go (TUG)

The Timed Up and Go (TUG) test [35] is a useful tool to assess gait-related functioning and motor symptoms because it involves sequential locomotor tasks that incorporate walking and turning, which are both affected by cognitive impairment [36,37]. In the assessment protocol, patients were seated on a chair, and were instructed to stand up, walk at their own comfortable and safe walking speed for 3 m, turn 180◦ at a designated spot, come back, and sit down on the chair again. The measured outcome is the time in seconds to complete the entire sequence. We used a digital sensor system developed in our laboratory for this purpose. Time started once the subject’s back left the chair and ended when the subject’s back touched the chair.

#### 2.3.5. Handgrip Strength Measurement

Maximal handgrip strength measurement was performed using a dynamometer Vernier with 1 Kh of sample rate. Patients were asked to squeeze the dynamometer with as much force as possible, not exceeding 5 s. Participants were asked (using a single question) whether they regarded themselves as left or right-handed. Two measurements were carried out for each hand, alternating the side, starting with the dominant hand. A rest period of approximately 60 s was used between trials. The unit of measurement was Newtons (N). The maximal strength from each hand was used in all analyses. The higher the value, the greater the strength of the individual [38].

### 2.4. Intervention

The intervention consisted in 24 sessions (3/week) of a cognitive-multisensory-physical exercise program carried out in the patient’s own home. The exercise program was designed by qualified personal trainers and psychologists, and implemented with the ROXPro© system (A-Champs) (https://a-champs.com; accessed on 8 January 2022). This system consists of small devices that provide visual, auditory and vibration stimuli with which the patient interacts. Through its mobile application, it allows the development of numerous sensory-cognitive-motor exercises adapted to the characteristics of the patients. In this study, the caregivers implemented the sessions previously designed by the therapists. Each session lasted approximatively 15 min. The difficulty of the exercises was increased across sessions, following the feedback provided by the caregivers and the application itself, which recorded the performance achieved by the patient on each exercise. Approximately 90% of the exercises included double task demands, while the rest consisted of simple walking exercises and reaching movements with the upper limb. Training adherence was reported as the percentage of training sessions performed by each patient.

## 3. Statistical Analysis

None of the data violated the normality assumption necessary to conduct parametric statistical tests according to the Shapiro-Wilk test.

To evaluate the effect of the exercise program in the variables recorded, we conduct several repeated-measures analysis of variance (ANOVA) with group (CG, EG) and time (Pre, Post) as the main factors. Post hoc *t* tests with Bonferroni corrections were computed when required.

*p*-values reported are based on two-sided comparison. A *p*-value lower than 0.05 was considered statistically significant. All statistical analyses were performed using SPSS (IBM, Chicago, IL, USA).

## 4. Results

Table 3 shows the main descriptive of the variables analyzed. With the exception of the dual task interference variable, the ANOVA did not show significant main effects or interactions for the other variables.

**Figure 2:**
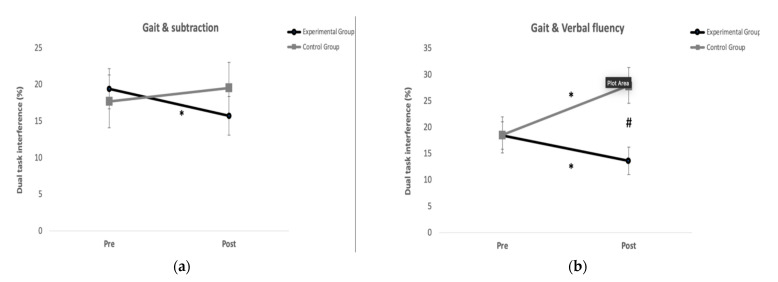
Results from the dual-task tests: (**a**) Experimental group reduced significantly the dual task interference for the Gait and subtraction test after the training program; (**b**) Experimental group reduced significantly the dual task interference for the Gait and subtraction test after the training program, while the control group increased it significantly. After the program training, the experimental group was significantly better than the control group in the Gait and Verbal fluency test. * Differences with pre (*p* < 0.05). ^#^ Differences with experimental group (*p* < 0.05).

The ANOVA showed significant interactions (group*time) for dual task interference during the subtraction condition (F = 4.81, *p* = 0.042; η^2^ = 0.22; observed power (OP) = 0.55) without significant group and time effects. Post hoc analysis indicated a significant reduction (*p* = 0.02) for the dual task interference only in the GE after the exercise program (Figure 2a). There were no significant differences between groups at Pre and Post evaluations.

The ANOVA showed significant interactions (group*time) for dual task interference during the verbal fluency condition (F = 16.50, *p* < 0.01; η^2^ = 0.49; observed power (OP) = 0.96) without significant group and time effects. The EG reduced significantly (*p* = 0.026) the dual task interference after the program exercise, while there was a significant increase (*p* < 0.01) in the CG. There was a significant difference (*p* < 0.01) in the dual task interference between groups at Post evaluation (Figure 2b).

## 5. Discussion

In the present study, we explored the effects of an exercise program characterized by combining physical, cognitive and sensory demands on dual-task gait performance in patients diagnosed with dementia. Our results suggest that the implementation of this program in the patient’s home and by relatives or caregivers is feasible and induces improvements in the performance of these tasks.

The subjects in the experimental group reduced the interference in the dual-task walking tests in the two cognitive task modalities after the exercise program; in contrast, the control group did not show such improvements, but an increase in interference with the verbal fluency task. Reduced interference in the dual-task walking test can be interpretated as an improvement in the performance of this task, since higher values of interference correlate with higher risk of fall episodes [32,39].

The reduction in dual-task interference cannot be the result of an improvement in gait performance, since there were no effects in the gait parameters during preferred and maximal speed in the experimental group. Up and Go performance did not change in either group, once again suggesting that the changes observed in the dual-task are not related to better gait or balance performance. There were also no significant changes in grip strength between the two tests. We included this test since a decreased hand motor function, similar to gait function, is also a possible candidate risk factor for cognitive impairment because of its association with cortical brain activity [40,41]. It is also unlikely that the short intervention period (24 sessions) and duration of each session (approximately 15 min) could have had a positive effect on any specific cognitive or executive functions that could explain the improvement in the dual-task walking performance, since long term and regular training is needed to effectively improve performance on executive functions (Baker et al., 2010) [42].

Although the interpretation of performance of dual-task walking is complex, we can speculate that the improvement in the performance of dual-task walking could have been the result of an improvement in the management of the attentional resources of these patients. Prioritizing balance or gait over other concurrent tasks is a common response in healthy young adults. However, several studies point out that this safe strategy is less frequent in older people [43]. In addition, subjects with some degree of cognitive impairment—such as that which may be present in PD, Alzheimer’s, or Progressive Supranuclear Palsy—would not show a clear choice of balance control over any other concurrent cognitive task. An incorrect task prioritization could lead these subjects to hazardous behaviours during dual-task conditions that require control of posture or gait, increasing the risk of falling [34]. Therefore, it is possible that participants in the exercise program acquired new strategies for dealing with dual-task situations designed by the therapists and incorporated into the exercise program. Let us highlight that the exercise program was made up mostly of exercises with dual-task characteristics that forced the patient to divide their attention during their execution and in which, through the ROX system, feedback could be given on which task to prioritize. Some of the tasks even implied three tasks simultaneously (i.e., the patient in standing position must move to step on a device located on the ground that emits a red colour while holding another device in his hand that vibrates if the position of the arm changes). Therefore, we suggest that improvements in dual-task performance were specific for the exercise program. This is in line with a previous study that showed a reduction in dual-task interference after a specific dual-task training in patients with dementia, without effects in cognitive domains other than the attention-related dual-task performances [44].

With the exception of one subject all participants completed the intervention program showing a high adherence to it. However, it is important to highlight that despite the individual sessions being carried out in the patient’s own home through the electronic device and its mobile application, there was remote support from the trainers, which probably contributed to the adherence to the program [25].

This is a pilot study and is therefore characterized by several limitations. First, the heterogeneity of the participants, not only at the level of cognitive impairment but also the different diagnoses, makes it difficult to extrapolate our results to a specific type of patient. However, this heterogeneity is representative of the diversity of patients that constitute Alzheimer’s associations, so any type of intervention to be carried out in or through them must be focused on individual characteristics. Second, in some subjects there were certain inconsistencies between the diagnosis and level of dementia and the scores reported on the scales. We must clarify that the researchers did not apply these scales. The diagnoses of dementia were made by neurologists, while the scales were applied by different neuropsychologists belonging to different local associations. Although the diagnosis of dementia was strictly medical, the inconsistencies in the scores on the scales may reflect different application criteria by these professionals. In fact, and in verbal communication, this issue was pointed out by one of the neuropsychologists. In addition, there is a lack of biomarkers in the medical diagnosis of dementia that would have been of interest to establish a correlation with the improvements observed in the dual-task. Therefore, this is another important aspect to take into account when carrying out studies with patients from different associative centers. Third, the reduced sample obtained for this pilot study clearly affects its statistical power. A possible cause for participating in the study could be the digital barrier, one of the main impediments to accessing this type of intervention [25,45]. However, it is not likely that this was the main cause, since the management of the electronic devices and their mobile application were simplified and there was daily remote assistance on demand from the patient. In interviews and informal chats held with members of Alzheimer’s associations, caregiver overload was the main argument for not wanting to participate in the study. This is a critical aspect to take into account when offering exercise programs to these groups. Finally, the assignment of subjects was made according to the disposition of the relatives or caregivers of the participants to implement the intervention program at home. This introduces a clear bias to be taken into consideration in future clinical studies. Therefore, one strategy would be to carry out a waiting list design only with patients whose relatives or caregivers are willing to participate.

## 6. Conclusions

In summary, our pilot study shows that the implementation of a home exercise program carried out with mobile technology in people with dementia is feasible and positively affects performance on dual tasks. These findings are relevant since they would allow us to respond to the challenges of implementing individual interventions in a real context.

## Figures and Tables

**Table 1:** Individual characteristics of the participants.

Diagnostic	Age	Sex	MMSE	IADL	BI
Vascular dementia	66	M	30	4	100
Parkinson’s disease	74	M	24	3	95
Alzheimer’s disease	69	F	18	3	100
Parkinson’s disease	74	M	28	4	95
Alzheimer’s disease	77	M	30	5	100
Alzheimer’s disease	74	M	25	8	100
Mild cognitive impairment	68	F	19	8	100
Parkinson’s disease	69	M	22	7	90
Lewy body dementia	72	M	23	5	90
Alzheimer’s disease	79	M	16	2	85
Alzheimer’s disease	77	M	20	3	100
Alzheimer’s disease	76	M	21	2	85
Alzheimer’s disease	79	M	26	8	100
Alzheimer’s disease	65	M	9	0	55
Parkinson’s disease	79	M	24	3	100
Mild cognitive impairment	79	M	17	5	100
Alzheimer’s disease	65	F	13	4	100
Parkinson’s disease	69	F	23	7	100
Alzheimer’s disease	70	F	22	5	95

Details of the total sample and the experimental and control groups are shown in Table 2.

**Table 2:** Characteristics of the overall sample and groups.

	Age	Sex	MMSE	IADL	BI
Overall sample (*n* = 19)	72 ± 4 (65–79)	14 males 5 females	21 ± 5.6 (9–30)	4 ± 2.25 (0–8)	93 ± 11 (55–100)
Experimental Group (EG) (*n* = 12)	72 ± 4 (66–79)	10 males 2 females	23 ± 5.36 (16–30)	4 ± 2.26 (2–8)	96 ± 5.39 (85–100)
Control Group (CG) (*n* = 7)	72 ± 7 (65–79)	4 males 2 females	18 ± 6.66 (9–24)	4 ± 2.64 (0–8)	91 ± 7.30 (55–100)

**Table 3:** Means and standard deviations in the performance of the tests before (Pre) and after (Post).

Task	Experimental Group		Control Group	
	Pre	Post	Pre	Post
Walking at preferred speed (m/s)	1.14 ± 0.14	1.07 ± 0.14	1.06 ± 0.25	1.05 ± 0.19
Walking at maximal speed (m/s)	1.66 ± 0.33	1.60 ± 0.36	1.56 ± 0.39	1.62 ± 0.27
Dual task interference (%) (Gait and subtraction)	19.41 ± 10.20	15.71 ± 9.36 *	17.68 ± 8.16	19.53 ± 8.88
Dual task interference (%) (Gait and Verbal fluency)	18.44 ± 9.29	13.61 ± 7.17 *	18.54 ± 8.51	27.01± 11.60 *^,#^
Time up and go test (s)	9.27 ± 1.59	9.51 ± 1.93	10.21 ± 2.07	9.62 ± 2.08
Grip strength dominant hand (N)	197.17 ± 61.74	200.34 ± 54.92	231.3 ± 97.65	226.78 ± 92.46
Grip strength non-dominant hand (N)	185.65 ± 55.85	193.67 ± 39.53	207.49 ± 127	208.53 ± 105.13

* Differences with pre (*p* < 0.05). # Differences with experimental group (*p* < 0.05).

## Data Availability

Data available upon request to the corresponding author.
